# Highly Stretchable Anisotropic Structures for Flexible Micro/nano-electrode Applications

**DOI:** 10.1186/s11671-016-1324-x

**Published:** 2016-02-29

**Authors:** Hao Guo, Jun Tang, Miaomiao Zhao, Wei Zhang, Jiangtao Yang, Binzhen Zhang, Xiujian Chou, Jun Liu, Chenyang Xue, Wendong Zhang

**Affiliations:** Science and Technology on Electronic Test & Measurement Laboratory, North University of China, Taiyuan, Shanxi 030051 China

**Keywords:** Flexible micro/nano-electrodes, Anisotropic structures, Highly stretchable, Strain sensitivity, PDMS

## Abstract

The functionality of flexible metal electrodes relies on the stable performance of a metallic film. In this study, highly stretchable anisotropic structures are designed to investigate the behaviour of silver electrodes on polydimethylsiloxane. Treated using both the oxygen plasma and the surface chemical functionalization technology, the film resistivity was reduced to 5.856 × 10^−9^ Ω m after annealing at 150 °C for 30 min, which is equal to that of commercial silver films on glass. The maximum variation in the resistance was approximately 5.315 % with a strain of 50 % along the four directions, and the films also show remarkable tolerance to repetitive strain. These results prove the excellent anisotropy performance of these structures to minimize resistive strain sensitivity and thus enable durable flexible electronics.

## Background

Recently, because of the major impact of devices using polyimide as a flexible substrate material and the associated need to define circuit paths for surface-mounted devices in mainstream electronics, which would enable the development of wearable electronic systems and highly sophisticated flexible mobile phones, flexible electrodes and wires have drawn increasing research attention [1–3]. Researchers have demonstrated a wide variety of technologies, including electronic skin [4], light-emitting diode (LED)-based semitransparent displays [5], and flexible thin film transistors [6] which have all been realized on fully flexible substrates.

To optimize the mechanical structures required and increase both flexibility and stretchability, many different materials and structures have been designed and studied [7]. Sekitani T et al. [8] proposed rubber-like stretchable elastic conductors based on single-walled carbon nanotube/polydimethylsiloxane (SWCNT/PDMS) composites that were integrated with a printed organic transistor active matrix. This material demonstrated good conductivity of 57 S/cm and stretchability of 134 %. Gutruf P et al. [9] designed stretchable micro-electrodes by controlling their sensitivity using serpentine designs and encapsulation. The resistance variation was approximately 22 % when the encapsulated 120° serpentine pattern was under a strain of 3 %. This method has been verified as a suitable way to manufacture micro-electrodes, but it proved not to be an efficient method for use in other engineering applications. Hong et al. [10] reported the formation of lateral-crack-free silver electrodes on highly pre-stretched PDMS grating substrates using the inkjet printing method followed by an annealing process while maintaining the pre-stretched state. The change in resistance of the fabricated silver electrodes under strains of up to 17 % was negligible. However, the electrodes using this structure can only be stretched in one direction for the pre-strain and are not suitable for use in practical applications.

To realize highly flexible electronic devices that operate under stress, a fundamental understanding of metalized polymer films and their behaviour under loading is extremely important. Also, to ensure that a working device is not affected by movement or deformation of the substrate, the resistive strain sensitivity of the materials must be reduced dramatically.

In this study, a novel stretchable isotropic structure has been designed to act as a micro/nano-electrode or wire structure that shows minimal change in its resistance when subjected to strain/stress. The highly stretchable wrinkled structure, which has been treated using both O_2_ plasma and sodium dodecyl sulphate (SDS) solution methods, has been processed to produce high adhesion between the surfaces to prevent the metal from slipping off the flexible substrate. Therefore, there is no cracking or deformation of the metal bonding to the wrinkled structure when the micro/nano-electrodes are subject to stretching, bending, or even twisting. The prevalent material combination of silver as the conductor and PDMS as the flexible substrate has been chosen to explore the possibilities for development of strain-independent micro-electrode conductive wires. Silver, which is a commonly used conductor in flexible and microelectromechanical structure (MEMS) devices, has been used and studied extensively [11]. Prior research has shown that silver provides excellent conductivity and extensibility for use in this work.

## Methods

**Fig. 1:**
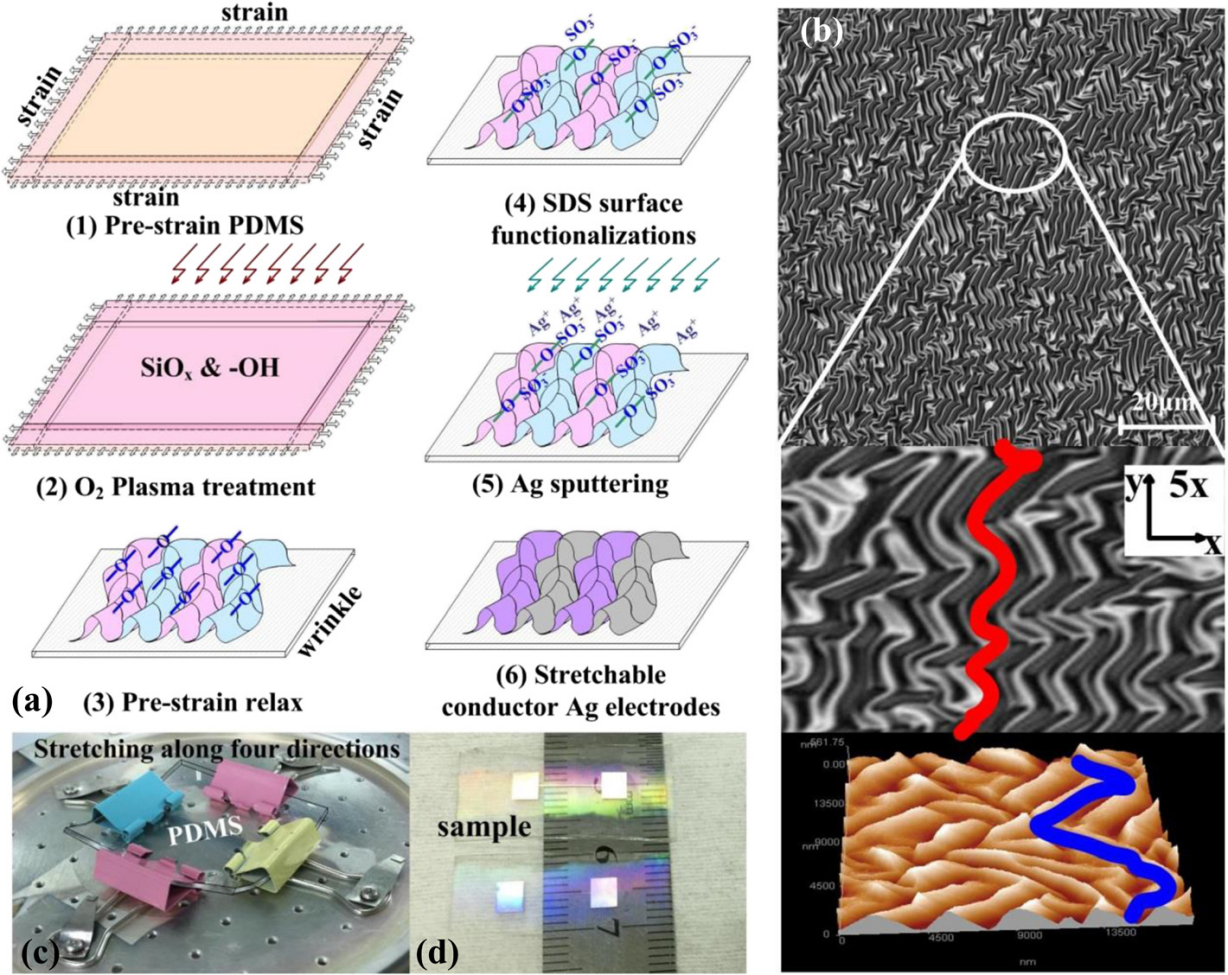
Fabrication process and morphology characterizations of the stretchable Ag electrodes. **a** PDMS treatment and Ag film deposition. **b** The shape of ‘S’ like of the electrode pattern from the laser confocal image and atomic force microscopy. **c** The PDMS substrate under pre-strained along the four different directions. **d** Optical images of the deposited wrinkled electrodes

Well-defined electrode wires with a width of 100 μm and length of 10 mm (as shown in Fig. 1d) were coated on the wrinkled PDMS films to study their strain sensitivity by subjecting these wires to constant loading and release. During the load–release cycling of the substrates, the resistance is measured constantly in situ.

The sinusoidal morphology with gradient periodicity of optical grating was formed on PDMS films. The PDMS films were prepared by mixing 12 g of pre-polymer (Sylgard184 from Dow Corning) with a curing agent in a 10:1 ratio using a spin coating process and cured at 80 °C for 2 h in the clean room. PDMS substrates with a thickness of 500 μm were prepared by controlling the spinning speed. The reference glass slide was thoroughly cleaned to prevent contamination.

The silver electrodes were then deposited on the PDMS substrates using a high-vacuum system (QPrep400, Mantis, England) by standard micro-fabrication techniques. The 100-nm-thick Ag films were grown under the following conditions: DC power of 45 W, Ar flow rate of 30 sccm, chamber pressure of 7.5 × 10^−3^ Torr, and process time of 3 min. All experiments were performed in a clean room area at a constant temperature of 20 °C with constant relative humidity of 60 %. The surface geometry of the grating arrays was characterized using atomic force microscopy (AFM, CSPM5000, Being Nano-Instrument Ltd., China) and laser scanning confocal microscopy (LSCM, OLS4100, Olympus, Japan).

## Results and Discussion

### Surface Functionalization and Ag Film Deposition

#### Fabrication of Ag Stretchable Electrodes

As shown in Fig. 1, the PDMS substrate has been first pre-stretched along the four directions (as shown in Fig. 1c). Then, the sample has been treated by oxygen plasma to form a SiO_x_ layer and hydrophilic groups (e.g. –OH) on the surface of the PDMS substrate as reported in our previous work [11]. The oxygen plasma attacks the siloxane backbone of PDMS, enhancing the interaction at the interface, to make single molecules more likely to spread onto the surface and form an oxygen-rich SiO_x_ silica-like layer and Si–OH compounds on the surface. When compared with the SiO_x_ layer with its higher modulus of elasticity, the PDMS material would require greater deformation to compress the SiO_x_ layer and form folded grating structures after strain relaxation [12].

The sample was then immersed in a SDS solution (with a concentration of 0.5 % SDS) for 15 s to introduce –SO_3_^−^ groups at the surface of the wrinkled PDMS grating. This ensured tight contact between Ag^+^ and PDMS through condensation reactions of the hydrophilic functionalities, which prevented the silver film from slipping away from the PDMS substrate under applied stress/strain. SDS is an anionic surfactant with amphiphilic properties because its molecular formula is CH_3_–(CH_2_)_10_CH_2_O–SO_3_–Na, which is composed of a lipophilic group of ethyl groups, CH_3_–CH_2_–, which reacts easily with Si–OH to form Si–O–SO_3_Na chains at the surface of the wrinkled PDMS, as shown in Fig. 1a (4). Because the hydrolyzation of the sulphate ion –O–SO_3_Na is performed under conditions of no catalyst and at normal temperature, the PDMS becomes permanently hydrophilic. The role of SDS as a functionalization agent for PDMS has been discussed in the literature [13, 14]. The sample was subsequently annealed at 150 °C for 30 min to improve both the quality and the conductivity of the silver films on the PDMS substrate.

Our method first uses oxygen plasma to treat the PDMS, followed by a second modification using a surfactant to overcome the drawback of the recovery phenomenon which implies that the PDMS surface recovers its hydrophobicity a few days after exposure to air when it has only been treated using the plasma technology [15]. This method uses plasma treatment to improve the activation properties of the inert polymer, forming rich Si–OH compounds that produce a condensation reaction with the SDS chain to render the PDMS surface permanently hydrophilic.

As it is shown in Fig. 1b, the isotropic electrode structure has S-like periodic grating structures, which have been tested by laser scanning confocal microscopy and atomic force microscopy. The ‘S’ shape can be stretched in any direction without tearing, and consequently, this is an important mechanism for the manufacture of stretchable electrode structures.

### Adhesion Testing

**Fig. 2:**
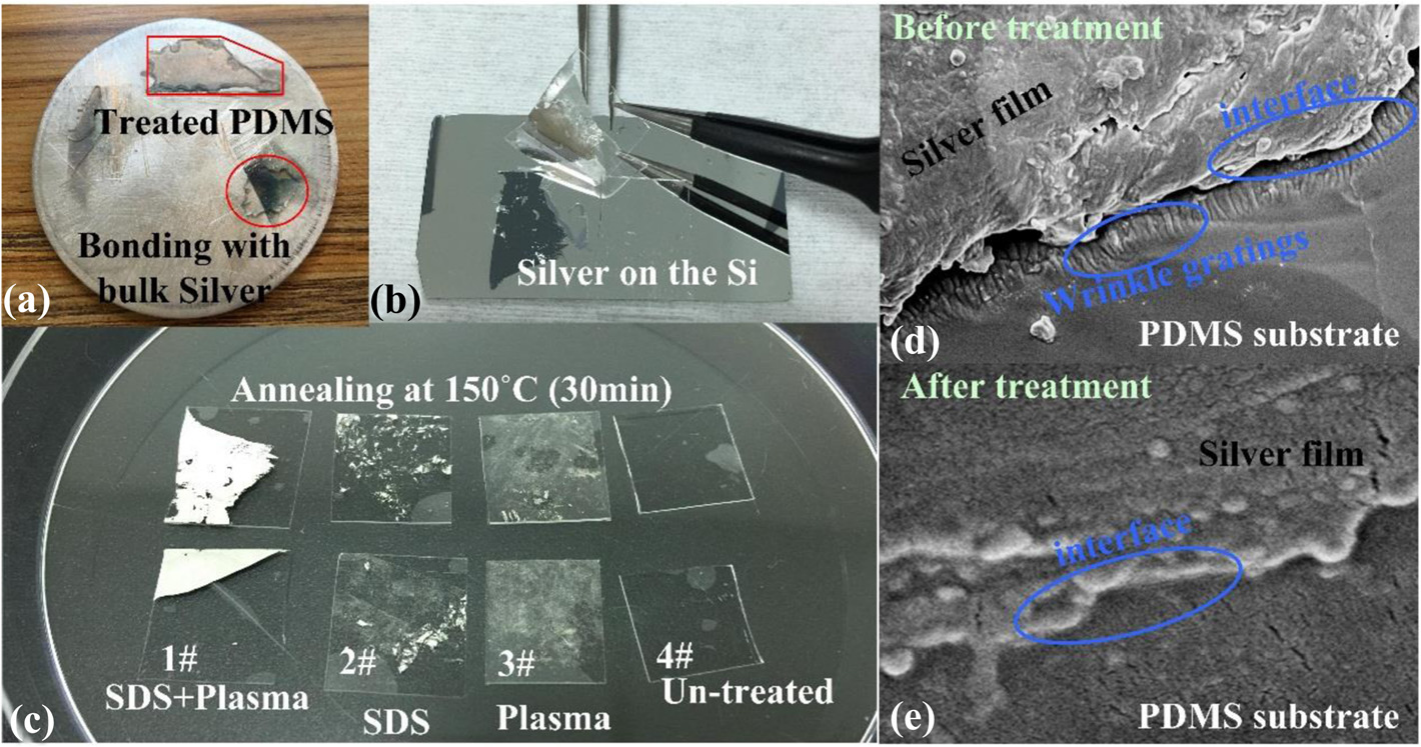
Adhesion test of the treated PDMS surface: bonding force characterizations of the PDMS film with chemical surface functionalization with different materials. **a** Bulk silver. **b** Bonding sliver from the Si using the treated PDMS. **c** PDMS treated by different methods. **d** Silver film on the PDMS substrate before treatment. **e** Silver film on the PDMS substrate after treatment

Bonding tests have been performed to characterize the film adhesion after the surface modification treatments. As shown in Fig. 2, visual modification effects have been observed. The silver film is first deposited on a silicon surface, which is to be used for debonding testing using PDMS samples for the debonding process. These samples have been previously surface-treated differently in order to compare the effect of each treatment on surface adhesion between Ag films and PDMS (as shown in Fig. 2a).

The PDMS samples were treated either with plasma or SDS only or with plasma and SDS combined, while an untreated PDMS sample (sample 4#) was used as a reference. Comparison of the four samples shows that the combined plasma and SDS method debonds the most silver from the silicon surface, i.e. the proposed method gives the best results, as it is demonstrated by sample 1# in Fig. 2b. Without the treatment, the silver film separated from the wrinkled grating on the surface of the PDMS substrate, and the silver film was observed to form multilayer and multi-aperture structures on the body, as shown in Fig. 2d. However, after treatment by the combined method of plasma and SDS followed by annealing at 150 °C for 30 min, almost the entire silver film is removed by the PDMS sample, while the adhesion to PDMS is apparently weaker in the other three cases (as shown in Fig. 2b). The corresponding section image explains this behaviour; as shown in Fig. 2e, based on the disappearance of the multi-layered and multi-aperture structures from the body of the silver film, the film has become smooth and dense, and the interface between the silver film and the wrinkled grating has bonded and been fused together. The contribution of the aggregation of the silver nanoparticle film into compact bulk silver material [16] is promoting the reaction of the –SO_3_^−^ group on the PDMS surface with more Ag^+^ dangling bonds and thus the formation of more and stronger chemical bonds between –SO_3_^−^ and Ag^+^. Using this method, the results are as shown in Fig. 2c, where the treated PDMS has been bonded with the bulk silver metal. This provides sufficient adhesive force between silver and PDMS to prevent the silver film from slipping away from the wrinkled PDMS grating and thus provides a novel and efficient method to protect the silver films from cracking during stretching, bending, or twisting.

### Ag Electrode Patterning and Characterization

To provide results for direct comparison to the PDMS samples, a static reference sample was also produced on an analogy glass slide on which a 100-nm-thick layer of silver was deposited using magnetron sputtering technology. Ag electrodes and wires were patterned using a shadow mask with a minimum line width of 100 μm using the same hardware to ensure process comparability.

Static tests were then conducted to determine the initial resistance values of these structures and the quality of the silver layers that had been treated by the various methods. A calibrated four-point probe measurement setup was used to determine the sheet resistance of the reference sample and that of the silver film on the PDMS substrate. The resistance measurements of the micro-scale electrodes were performed using an Agilent 4156C parameter analyser after the wire resistance of the measurement equipment was taken into account.

**Fig. 3:**
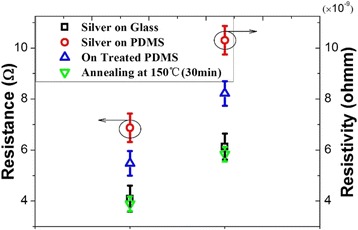
Static measurements of the reference silver conductivity lines on glass and the silver lines subject to test on treated PDMS by different methods vs. theoretical resistivity from four-point probe measurements

Figure 3 shows the sheet resistance of the silver film on glass to be 4.18 Ω, and a corresponding resistivity of 6.271 × 10^−9^ Ω m was calculated using the equation *ρ* = *R* × *S*/*L*. This value shows good agreement with the values that were reported in the literature for thin gold films on glass [17]. Interestingly, a higher sheet resistance (6.87 Ω, with a corresponding resistivity of 10.305 × 10^−9^ Ω m) is measured for the silver film that was deposited on the PDMS substrate without any treatment. Using the combined treatment method based on plasma and SDS technologies, the sheet resistance was reduced to 5.48 Ω and the corresponding resistivity was 8.221 × 10^−9^ Ω m. After annealing, the resistance reached its best value of 3.904 Ω, and the corresponding resistivity was 5.856 × 10^−9^ Ω m. As shown in Fig. 3, the measured values for the substrates produced by the different methods showed close agreement with the results of the theoretical calculations that were performed using the resistivity values obtained from the four-point probe measurements.

Dynamic measurements were then performed to characterize the resistive strain sensitivity of the silver electrodes of the wires on the PDMS. The test setup was conducted using a home-made fully automated stretching apparatus that was composed of a linear stage actuated using a micrometer screw that was driven by a stepper motor with a step resolution of 10 μm. The stepper motor was controlled using a LabView programme to control the sample displacement. The resistance was measured in precise synchronization with the displacement using the Agilent 4156C after each displacement step.

**Fig. 4:**
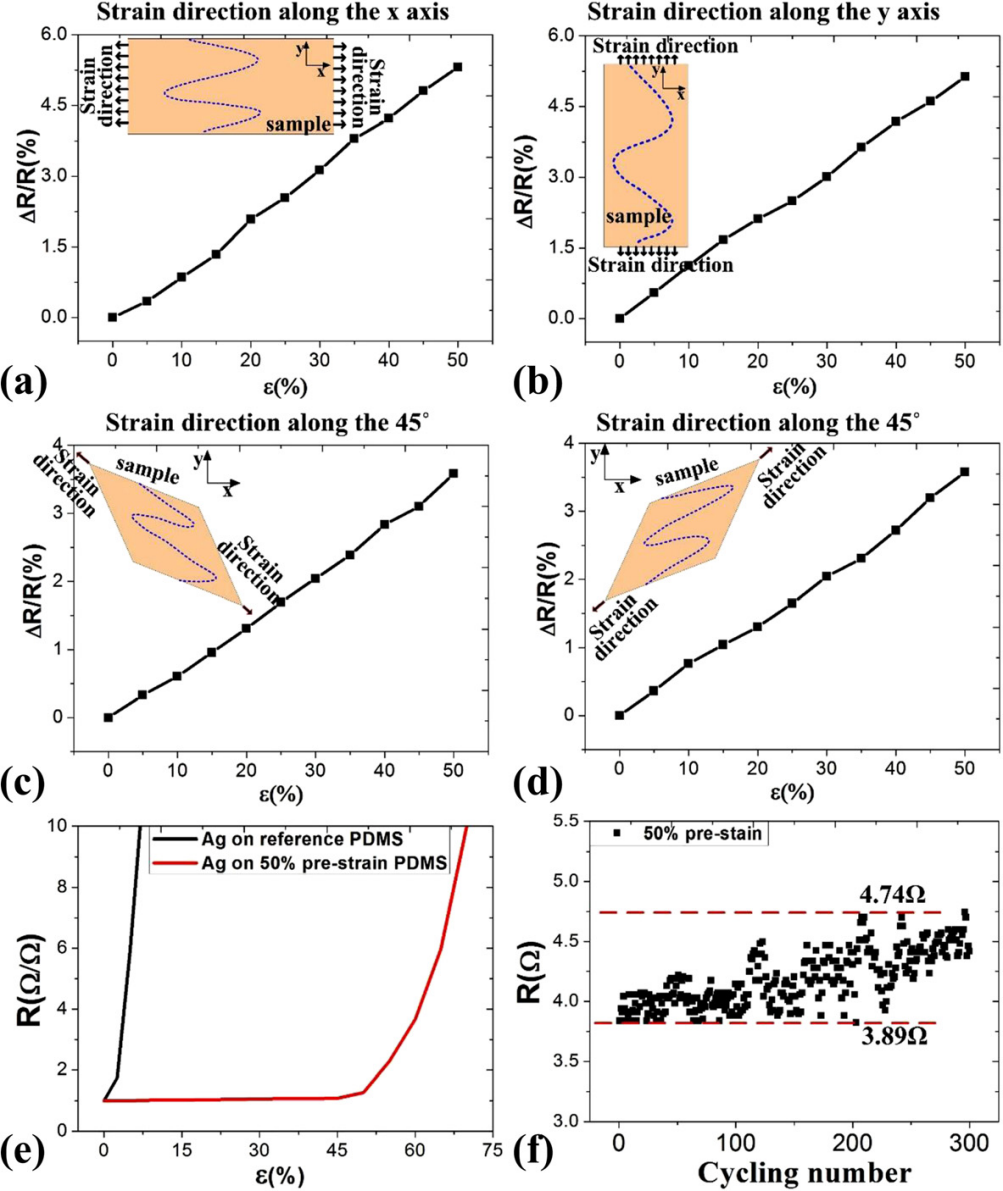
Resistive response characterizations of the wrinkled electrodes under the strain along four directions. **a** Along the *x*-axis. **b** Along the *y*-axis. **c** Along the oblique direction and 45°. **d** Along the opposite direction to **c. e** Normalized resistance versus applied strain. **f** Reproducibility characterization

For the isotropic stretching performance test, we were measuring the resistance of the silver electrodes on the wrinkled structure while a tensile stress was applied slowly. As it is shown in Fig. 4a, the resistance variation of the silver electrode was only 5.314 % when the applied strain was along the *x*-axis of the sample, while the resistance variation was 5.128 % when the strain was applied along the *y*-axis (as shown in Fig. 4b). When the strain was applied along the clinodiagonal direction of the sample (i.e. at 45°), the observed changes in the resistance of the silver electrode were approximately 3.585 and 3.573 % (as shown in Fig. 4c, d, respectively). The changes along the clinodiagonal direction were smaller than those that occurred along the *x*- and *y*-axes, because stress along the clinodiagonal direction can be decomposed in both the *x*- and *y*-axes, and the sum of the stress in *x*- and *y*-axes was less than 50 % because of the Poisson effect. The reason for this behaviour is that when the sample has been stretched along the *x*-axis (producing the *x* component of the clinodiagonal stress), there was also a compressive deformation to offset the tensile deformation of the *y*-axis (the *y* component of the clinodiagonal stress).

Also, a theoretical estimate of the strain on the silver electrodes can be calculated during the process of stretching. The change in the resistance of an ideal straight silver conductor with a pure dimensional elastic change in shape can be estimated using the following equation [8]:

1 $$ \varDelta R={R}_0\frac{\left(1+{\varepsilon}_l\right)}{{\left(1+v{\varepsilon}_l\right)}^2} $$

Here, *R*_0_ was the initial resistance, *v* was the Poisson’s ratio of silver (0.37), and *ε*_*l*_ was the strain on the silver film.

Using Eq. (1), the strain on the silver electrode was determined to be less than 3 % for a change in resistance of 5.31 %. This means that the strain on the silver electrode on the wrinkled structure was only 3 % when the sample was being stretched with a strain of 50 %. These wrinkled structures can thus offset the strain of stretching to ensure that the silver electrode remains conductive without any cracking. This proves that this structure can be used to manufacture stretchable electrodes for application under low- or high-tensile fields.

Figure 4e shows the variation in the normalized resistance for the silver electrode, which is the ratio of the measured resistance to the initial resistance. The silver film on the 50 % pre-stretched PDMS substrate showed much better stretching performance than that on the non-stretched PDMS surface (silver on reference PDMS). The silver electrodes formed on the wrinkled PDMS structure remain completely functional with deformation of the wrinkled structure during stretching. In comparison, the silver electrodes formed on not pre-strained PDMS became insulating under a very small amount of strain because of the formed micro-cracking of the silver film as a result of the applied stress.

Because they did not suffer any cracking, the silver electrodes on the wrinkled structure showed good initial conduction properties and maintained these properties during repeated stretching processes. As shown in Fig. 4f, these silver electrodes show negligible resistance change in the low-strain region. Therefore, using a sample with a pre-strain of 50 %, we performed a cycling test at 50 % of the maximum strain. The resistance increases slightly, from an initial resistance of 3.89 Ω to a final resistance of 4.74 Ω, after 300 stretching cycles. This result clearly shows that our structures can sustain their initial conductivity under repeated stretching conditions. During tensile processes, the Ag films do not slip or crack, leading to conductance stabilization, because of the strong adhesion between the silver film and the PDMS wrinkled grating structure.

### Demonstration of Application Examples

As a proof-of-concept for stretchable electronics applications, we have used the as-prepared silver stretchable electrodes as connecting wires to power a commercial LED with a turn-on voltage of 2.5 V.

**Fig. 5:**
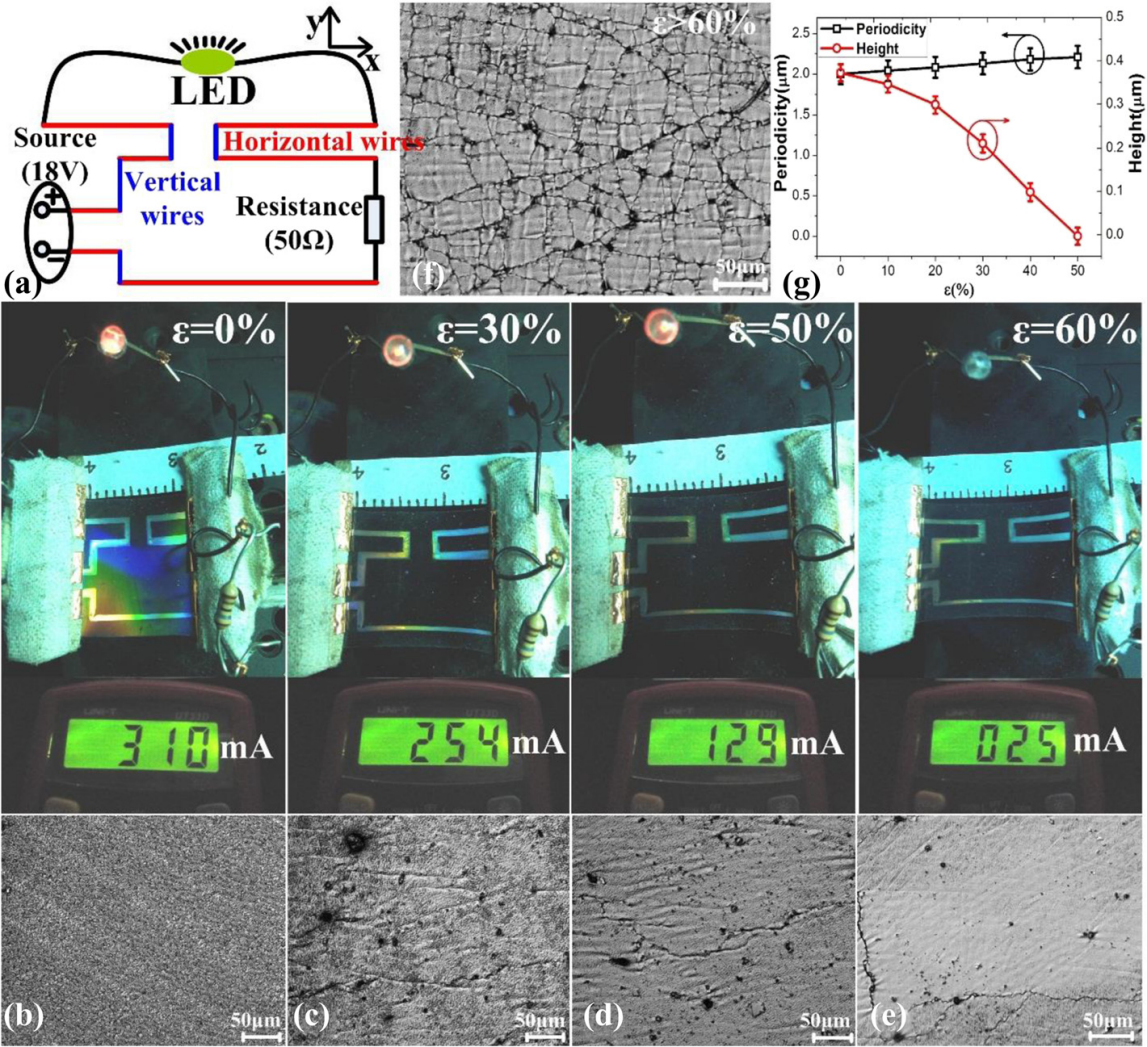
Electronic circuit application test with vertical and horizontal conductive wire in length of 500 μm. **a** The schematic diagram using the horizontal and vertical conductive wires based on anisotropic wrinkle gratings. **b**–**e** The relative changes of light intensity of LED with the corresponding electric current and the microscopic morphological characteristics under increased strain of 60 % along the *x*-axis. **f** The corresponding microscopic morphological characteristics after the strain was more than 60 %. **g** The periodicity and height of wrinkle grating structures under the strain

As shown in Fig. 5a, the silver electrode wire mapping has been designed as zigzag lines at a vertical angle to verify the isotropic properties of our wrinkled structures on PDMS with a pre-strain of 50 %. The red lines represent the horizontal silver electrode wires along the *x*-axis, and the blue lines represent the vertical silver electrode wires along the *y*-axis. Under stretching along the *x*-axis, the red silver electrode wires were stretched in length along the applied strain direction, while the blue wires were stretched in width, perpendicular to the applied strain.

As shown in Fig. 5b–e, when the strain increased from 0 to 30 %, or even as high as 50 %, the LED was still lit, although the corresponding electric current decreased from 320 mA to 254 mA and ultimately to 129 mA. This is a proof that none of the silver electrode wires were cracked or broken under a strain of less than 50 %. However, when the strain increased to 60 %, the LED did not operate because the corresponding electric current had been reduced to 25 mA, which was too low to turn the LED on. This is attributed to the silver electrode that had begun to be flattened and to break up with increasing numbers of cracks, as shown in the corresponding microscopic morphological characteristics in Fig. 5b–e. Under the increased strain, the wrinkled grating structure had been stretched out to become flat and thus had begun to crack after the grating height decreased to zero (as shown in Fig. 5g) under an applied strain that was higher than the pre-strain. These results demonstrate that our wrinkled design is a highly stretchable isotropic electrode structure with a great potential for further exploration in application fields such as bionic skin and flexible electronic devices.

## Conclusions

We have examined the isotropic properties of silver micro-electrodes on a pre-strained and relaxed PDMS substrate, which was fabricated after the deposition of silver on the wrinkled PDMS, which was initially treated using O_2_ plasma followed by surface functionalization using SDS to ultimately form well-adhered silver electrodes with a linewidth of 100 μm.

These static silver films were characterized by four-point probe measurements to determine the resistivity of the silver films that had been treated using various methods. After treatment with the combined plasma and SDS method followed by annealing at 150 °C for 30 min, the resistivity of the silver electrode on the wrinkled PDMS was reduced to 5.856 × 10^−9^ Ω m, which was equal to the resistivity of commercial silver on glass.

We also evaluated the isotropic behaviour of these silver electrodes when they were subjected to strain. The resistance variations of the silver electrodes on the wrinkled PDMS were as low as 5.314 % along all four directions when the strain reached a value of 50 %. It was also shown that these silver stretchable electrodes could feasibly be used as connecting wires for stretchable circuits. The silver electrode wire mapping circuits were designed as zigzag lines at a vertical angle to prove the isotropic properties of our wrinkled structures. These findings highlight the significance of these isotropic properties for durable flexible electronics, which can be widely used, not only as interconnects but also as electrodes for flexible electronics technologies, including electronic skin and flexible display technology.
